# Dr. Electa B. Whipple

**Published:** 1907-10

**Authors:** 


Dr. Electa B. Whipple, of Buffalo, died at her summer home at Orchard Park, September 13, 1907. She was born at Perrysburg, N. Y., and received her preparatory training first at Gowanda, then at Lima Seminary, and finally at Syracuse University, where she received the degree of A.B. in 1874. After graduating in medicine in 1884, at Syracuse University, she spent some time at Attica associated in practice with Dr. Davis. She came to Buffalo about twenty years ago and had been engaged in practising her profession in this city until her death. Dr. Whipple was one of the best known women physicians in Buffalo and was a prominent member of several medical societies.

